# A Consistent Landmark for Tibial Tunnel Placement in Arthroscopic Remnant-Preserving Posterior Cruciate Ligament Reconstruction: Use of Champagne-Glass Drop-Off and Lateral Cartilage Point—A Retrospective Case Series

**DOI:** 10.3390/diagnostics16111688

**Published:** 2026-05-29

**Authors:** Yu-Ze Luan, Wei-Jun Hong, Tzu-Chun Chung, Chien-Sheng Lo

**Affiliations:** 1 School of Medicine, Chung Shan Medical University, No. 110, Section 1, Jianguo North Road, Taichung City 40221, Taiwan; yuzeluan2002@gmail.com; 2 Department of Orthopedic Surgery, Kuang Tien General Hospital, No. 127, Xiangshang Road, Shalu District, Taichung City 43303, Taiwan; staylast@gmail.com; 3 Department of Education, China Medical University Hospital, No. 91, Xueshi Road, North District, Taichung City 404328, Taiwan; chung24755@gmail.com; 4 Department of Orthopaedics, Chung Shan Medical University Hospital, No. 110, Section 1, Jianguo North Road, Taichung City 40221, Taiwan; 5 Institute of Medicine, Chung Shan Medical University, No. 110, Section 1, Jianguo North Road, Taichung City 40221, Taiwan

**Keywords:** remnant-preserving posterior cruciate ligament reconstruction, tibial tunnel placement, transseptal portal, champagne-glass drop-off, lateral cartilage point

## Abstract

**Background/Objectives:** Accurate tibial tunnel placement is critical for successful posterior cruciate ligament reconstruction (PCLR), yet remains technically demanding due to limited visualization and anatomic variability. This study aimed to demonstrate the feasibility of an arthroscopic technique for remnant-preserving PCLR using the champagne-glass drop-off and lateral cartilage point as consistently identifiable arthroscopic anatomic bony landmarks, and to evaluate the success rate of tibial tunnel placement in targeted position using postoperative magnetic resonance imaging (MRI). **Methods:** A retrospective review was performed of patients who underwent arthroscopic remnant-preserving PCLR using a trans-septal approach with the described dual-landmark technique between March 2020 and October 2022. Of 31 eligible patients, 20 with complete clinical follow-up and postoperative 1-year MRI were included for analysis. Tibial tunnel position was assessed on MRI to determine success rate of placement in the targeted inferior–lateral tibial footprint based on anatomic reference. Clinical outcomes, including knee range of motion and posterior laxity, were also evaluated. **Results:** MRI evaluation demonstrated tibial tunnel consistent placement with the predefined targeted zone in all patients (20/20). At a median follow-up of 745 days, the mean knee range of motion was 140.0 ± 12.7 degrees. Posterior stability assessment showed grade 0 laxity in 75% of patients and grade 1 laxity in 25%. No graft failures, neurovascular complications, infections, or revision PCLR procedures were observed. **Conclusions:** This retrospective case series suggests that the dual-landmark technique (champagne-glass drop-off and lateral cartilage point) may facilitate consistent tibial tunnel placement in remnant-preserving PCLR. Level of Evidence: IV (Retrospective case series).

## 1. Introduction

Posterior cruciate ligament (PCL) injuries are substantially less common than anterior cruciate ligament (ACL) injuries, accounting for approximately 0.65% of sports-related knee injuries compared with 20.3% for ACL injuries in a large epidemiologic series [1]. Nevertheless, inadequately treated PCL injuries are frequently associated with persistent pain, functional instability, and progressive degenerative changes of the knee joint. Clinical evidence has demonstrated that untreated or non-operatively managed PCL injuries are associated with a relatively high incidence of secondary osteoarthritis, reported in up to 44% of patients at mid- to long-term follow-up, compared with approximately 22% following surgical reconstruction [2]. In addition, residual posterior tibial laxity may persist, contributing to altered knee biomechanics and functional impairment. While conservative management may be appropriate for selected low-grade injuries, surgical reconstruction is generally indicated for patients with high-grade PCL injuries (e.g., Hughston grade II-III) or combined ligamentous injuries to restore posterior stability and knee biomechanics [2,3]. Despite continuous refinement of surgical techniques, posterior cruciate ligament reconstruction (PCLR) remains technically demanding because the tibial PCL footprint is located deep within the posterior compartment, where arthroscopic visualization is limited, and tunnel placement must be performed in proximity to the popliteal neurovascular structures. PCLR is also associated with higher failure rates than ACL reconstruction, with reported rates exceeding 20%, predominantly due to residual posterior laxity [4]. Among the multiple determinants of surgical success—including graft choice, fixation strategy, and postoperative rehabilitation—accurate anatomic placement of the tibial tunnel has consistently been identified as the most critical factor influencing clinical and biomechanical outcomes [5].

**Figure 1:**
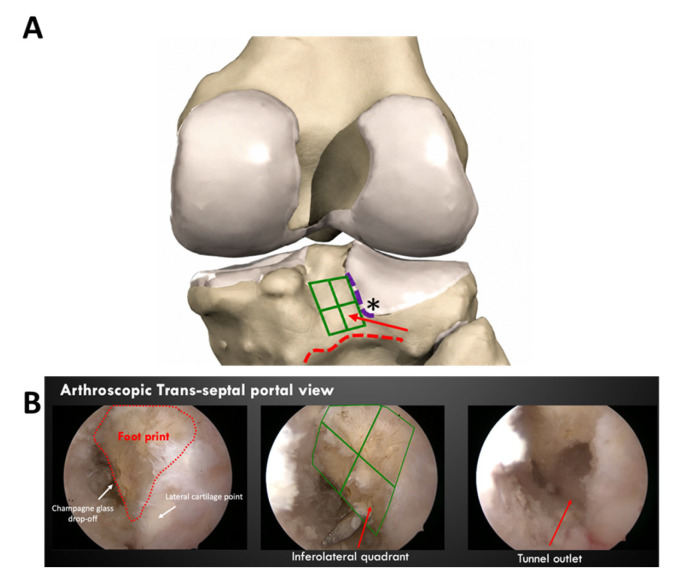
Anatomical landmarks and tibial tunnel placement in remnant-preserving posterior cruciate ligament reconstruction. (**A**) The tibial footprint of the posterior cruciate ligament is divided into four quadrants by the green lines. The red arrow indicates the inferolateral quadrant. The red dashed line represents the champagne-glass drop-off, the purple dashed line represents the lateral cartilage border, and the asterisk indicates the lateral cartilage point. (**B**) Arthroscopic trans-septal view of the tibial footprint. The complete tibial footprint is outlined by the red dashed line in the left panel. The tibial tunnel is positioned within the inferolateral quadrant, as indicated by the red arrow in the middle panel. The tunnel outlet is indicated by the red arrow in the right panel.

From an anatomic and imaging perspective, the tibial insertion of the PCL is located deep within the posterior intercondylar fovea, a region that is difficult to visualize intraoperatively and exhibits substantial individual variability. Even minor deviations from the ideal tunnel position may substantially alter graft biomechanics, increase stress concentration at the tibial “killer turn,” and predispose to graft elongation or failure [6,7]. Imaging-based and biomechanical studies have demonstrated that optimal tibial tunnel placement is located within the inferolateral quadrant of the native PCL footprint, which minimizes posterior tibial translation and optimizes graft tension patterns [8] (Figure 1).

Intraoperative fluoroscopy has traditionally been used to assist tibial tunnel positioning; however, this modality exposes patients and surgical staff to ionizing radiation, prolongs operative time, and may be limited by projection errors related to tibial rotation or overlapping bony structures [9]. Advances in arthroscopic techniques, particularly the trans-septal portal approach, have improved direct visualization of the posterior tibial plateau. Nevertheless, precise and reproducible identification of the ideal tunnel location remains challenging, and significant variability persists among surgeons.

Several arthroscopic landmark-based techniques have been described, including palpation of the foveal borders, placement relative to the tibial joint line, or measurement from the posterior tibial cortex [9,10,11]. However, many of these methods rely on soft tissue references that may be distorted by synovial hypertrophy, scar formation, or the PCL remnant, thereby reducing reliability and interobserver reproducibility. In contrast, bony landmarks provide fixed reference points that are independent of soft tissue conditions.

The “champagne-glass drop-off,” a characteristic cortical contour of the posterior tibia, has been described as a useful sagittal-plane landmark for tibial tunnel positioning and can be identified under trans-septal arthroscopic visualization based on prior reports [8]. However, a validated and reproducible coronal-plane (medial–lateral) bony landmark under arthroscopic visualization has been lacking, contributing to variability in tunnel placement along this axis. The “lateral cartilage point,” corresponding to the lateral boundary of the PCL tibial footprint, has been proposed as a potential coronal-plane landmark and is detectable arthroscopically [12]. Importantly, identification of this structure through conventional anterior portals often necessitates extensive debridement of native PCL fibers, which may compromise remnant preservation.

Recent imaging and clinical studies have increasingly emphasized the diagnostic and biological importance of remnant-preserving PCLR, demonstrating improved graft maturation, proprioceptive preservation, and favorable radiological and clinical outcomes [13,14,15,16,17]. Preservation of native PCL tissue has also been shown to facilitate more accurate anatomical orientation during reconstruction.

To address the limitations of existing techniques, we developed a dual-landmark arthroscopic approach that integrates the champagne-glass drop-off (sagittal plane) and the lateral cartilage point (coronal plane), both identifiable through trans-septal arthroscopy while preserving maximal PCL remnant tissue. Together, these landmarks define a consistently identifiable anatomic “target zone” for tibial tunnel creation. The study aimed to describe and evaluate this dual arthroscopic landmark technique for tibial tunnel placement in remnant-preserving PCLR. We hypothesized that this arthroscopic dual-landmark technique would facilitate consistent tibial tunnel placement, minimize intraoperative variability, and support anatomically appropriate remnant-preserving PCLR by combining complementary sagittal- and coronal-plane bony references to overcome the limitations of single-plane landmark guidance.

## 2. Materials and Methods

### 2.1. Patient Selection

This retrospective study reviewed consecutive patients who underwent single-bundle PCLR performed by a single orthopedic surgeon between March 2020 and October 2022 at Chung Shan Medical University Hospital, Taichung, Taiwan.

The inclusion and exclusion criteria were as follows.

Inclusion criteria:

- Patients who underwent remnant-preserving single-bundle PCLR;
- Availability of complete postoperative clinical follow-up;
- Availability of postoperative MRI.

Exclusion criteria:

- Incomplete clinical follow-up;
- Incomplete MRI follow-up.

**Figure 2:**
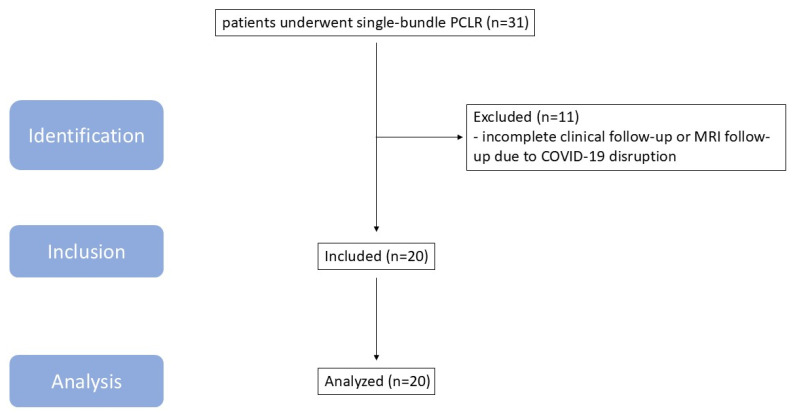
Flow diagram of patient selection. A total of 31 consecutive patients who underwent single-bundle PCLR were identified. After application of inclusion and exclusion criteria, 20 patients with complete clinical and imaging follow-up were included in the final analysis.

Of the 31 patients initially identified, 20 met the inclusion criteria and were ultimately included in the analysis, whereas the remaining patients were excluded due to follow-up disruptions during the COVID-19 pandemic. A flow diagram illustrating the patient selection process is provided in Figure 2. All surgical procedures were performed by the same surgeon using a standardized arthroscopic technique and uniform postoperative rehabilitation protocol. Demographic data, surgical records, imaging studies, and follow-up clinical assessments were reviewed. This study was conducted in accordance with institutional ethical guidelines.

### 2.2. Surgical Technique

**Figure 3:**
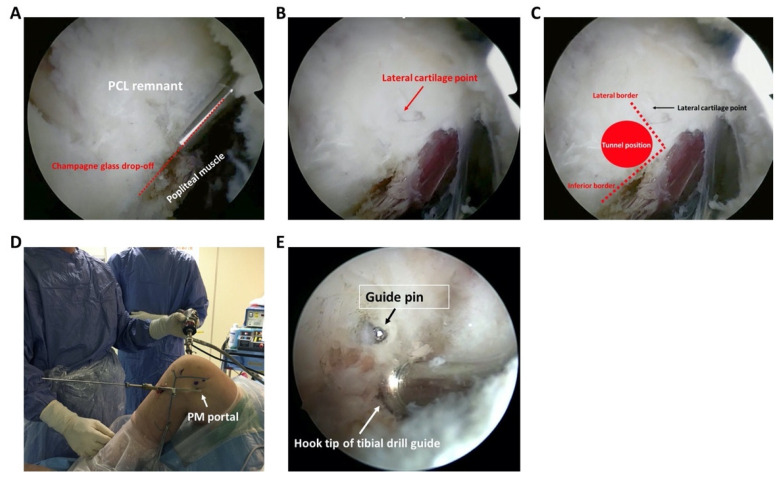
Arthroscopic images demonstrating the surgical technique. (**A**) trans-septal portal viewing from PM portal, Champagne glass drop-off (red dotted line); (**B**) the lateral cartilage point (red arrow) is regarded as lateral border of tibial footprint; (**C**) tunnel position (red circle) located between the “Champagne glass drop-off”—inferior border and the lateral cartilage point—lateral border; (**D**) tibial drill guide was introduced through PM portal; (**E**) the hook tip of drill guide was targeted to the position between inferior and lateral border.

The study cohort consisted of 20 patients (14 males and 6 females) with a mean age of 25.5 years (range, 16–41 years). This cohort included isolated, revisional, and combined PCLR cases, including concomitant anterior cruciate ligament reconstruction (ACLR), posterolateral complex reconstruction (PLCR), or high tibial osteotomy (HTO) (Table 1). In all cases, arthroscopic PCLR was performed using a remnant-preserving single-bundle technique without the use of intraoperative fluoroscopy. A trans-septal portal was routinely established to optimize visualization of the posterior tibial footprint while preserving native PCL fibers. The “champagne-glass drop-off” was first identified arthroscopically as the inferior bony margin of the tibial PCL footprint (Figure 3A). Following identification of this sagittal-plane landmark, the posterior capsule was progressively released in a lateral direction along the champagne-glass drop-off until the lateral cartilage point was visualized (Figure 3B), defining the lateral boundary of the tibial footprint. The region bounded by the champagne-glass drop-off inferiorly and the lateral cartilage point laterally was designated as the tibial tunnel target zone (Figure 3C). For transtibial tunnel creation, the hook tip of the tibial drill guide was introduced through the posteromedial (PM) portal (Figure 3D) and oriented toward the inferolateral aspect of the tibial footprint (Figure 3E), ensuring tunnel placement within the predefined target zone (Supplementary Video S1). The femoral tunnel was created using an inside-out technique, positioned between the anterolateral and posteromedial bundle footprints and approximately 3 mm from the articular cartilage margin. The graft type was selected according to patient factors, revision status, and graft availability. Autologous tendon grafts, including the peroneus longus tendon combined with the semitendinosus tendon when required, or allograft Achilles tendon were used. After harvest or preparation, the graft was folded to achieve an appropriate graft diameter for PCLR. The individual graft size was listed in Table 1. Graft fixation was achieved using a suspensory fixation device on the femoral side and aperture fixation with a bioabsorbable interference screw on the tibial side. Additional tibial fixation was reinforced using a screw-and-washer post on the proximal tibia.

### 2.3. Postoperative Rehabilitation

All patients followed an identical, standardized rehabilitation protocol under the supervision of a single licensed physical therapist. During the first postoperative month, the knee was immobilized in full extension. Beginning in the second postoperative month, closed-chain range-of-motion (ROM) exercises were initiated, with flexion limited to 90° for the first three months. The knee brace was discontinued after three months, and progressive ROM exercises were advanced to achieve full knee motion by postoperative months four to five. During the first postoperative month, patients were allowed partial weight bearing with crutch support. Full weight bearing was permitted beginning in the second postoperative month. Return to unrestricted daily activities was permitted at six months postoperatively, and return to sports activities was allowed at nine months.

### 2.4. Imaging Assessment and Clinical Evaluation

**Figure 4:**
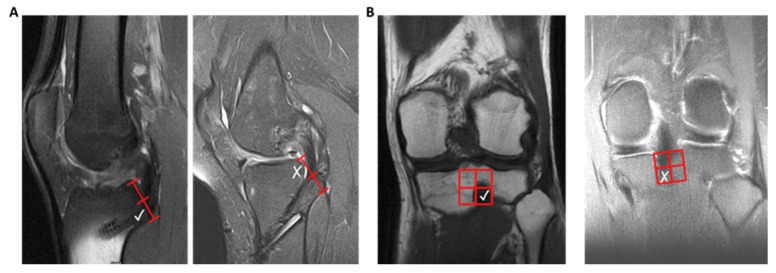
MRI images demonstrating the evaluation method of ideal tibial tunnel opening. (**A**) red line divided retro-spinal surface to superior and inferior half; **left**: ideal tunnel located in the inferior half; **right**: non ideal tunnel located in superior half; (**B**) Red frame demonstrated the coronal plane of tibial footprint, and its midline divided the lateral and medial half, **left**: ideal tunnel located in lateral half; **right**: non ideal tunnel located in medial half. The white check mark indicates ideal position, whereas the white cross symbol indicates non ideal position.

**Figure 5:**
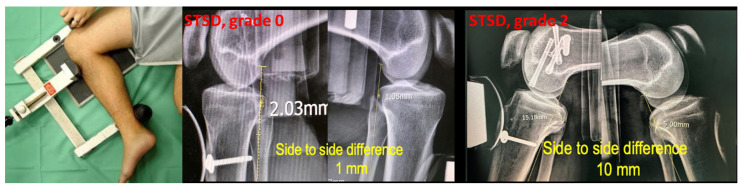
Assessment of posterior instability using lateral stress radiography. **Left** panel, the patient positioned in the lateral decubitus position for lateral stress radiographic assessment; posterior knee instability Grade 0, STSD in posterior tibial translation of 0–3 mm (**middle** panel) and Grade 2, 5–10 mm (**right** panel).

Postoperative MRI was performed approximately one year after surgery to assess tibial tunnel position and graft signaling. Computed tomography (CT) was precluded due to radiation exposure and insurance limitations in this cohort. Tibial tunnel position was evaluated on both sagittal and coronal MRI planes. On sagittal images, the tibial PCL footprint was divided into superior and inferior halves, with tunnel openings located within the inferior half considered ideal (Figure 3A). On coronal images, a vertical reference line passing through the center of the tibial eminence divided the footprint into medial and lateral halves; tunnels located predominantly within the lateral half were defined as optimal position (Figure 3B). MRI assessments were independently performed by two authors using standardized measurement criteria. At the time of image assessment, the evaluators were blinded to postoperative clinical outcome data. Clinical outcomes, including knee ROM and posterior stability, were evaluated during postoperative follow-up visits. Posterior instability was quantified using lateral stress radiographs (Figure 4), with posterior tibial translation graded according to the side-to-side difference (STSD). The STSD was calculated as the posterior tibial translation distance of the affected knee minus that of the contralateral knee. As shown in Figure 5, the patient was placed in the lateral decubitus position with the knee flexed at 90°. A posteriorly directed force was applied to the anterior aspect of the tibial tuberosity (TT), and a lateral radiograph was obtained. The STSD in posterior tibial translation between the affected and contralateral knees was measured radiographically. Posterior knee instability was graded according to the STSD as follows: Grade 0, 0–3 mm; Grade 1, 3–5 mm; Grade 2, 5–10 mm; and Grade 3, >10 mm.

## 3. Results

### 3.1. MRI-Based Assessment of Tibial Tunnel Position

Postoperative MRI demonstrated that all tibial tunnels were placed within the predefined target zone in all evaluated knees (20/20) according to the categorical assessment criteria in this study. Successful MRI-defined inferior and lateral tibial tunnel placement was observed in all 20 patients, corresponding to an observed success rate of 100%. The exact 95% confidence interval for this proportion was 83.2% to 100%, reflecting the limited precision of this estimate due to the small sample size. On sagittal-plane analysis, all tibial tunnel apertures were located within the inferior half of the tibial PCL footprint. Coronal-plane assessment similarly confirmed that all tunnel apertures were positioned within the lateral half of the footprint, corresponding to the predefined targeted anatomic zone. Independent evaluations performed by two investigators yielded identical findings. Both evaluators identified the tunnel positions within the same predefined categorical regions in all cases (Table 1).

### 3.2. Clinical Outcomes

**Figure 6:**
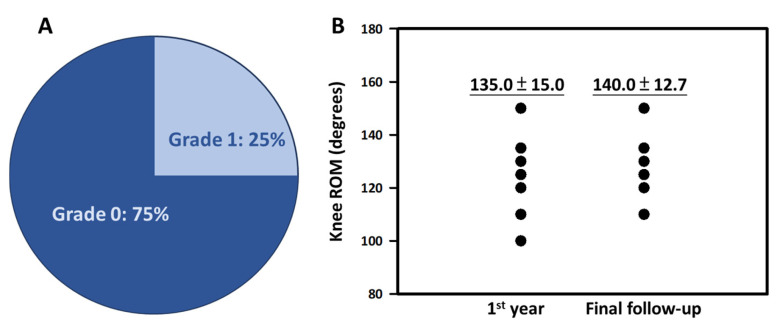
Postoperative rehabilitation. The patients (*N* = 20) were evaluated and documented at 1st year and final follow-up postoperatively. The clinical outcomes were represented as (**A**), joint laxity and (**B**), knee ROM.

The median postoperative follow-up duration was 745 days (interquartile range, 640–850 days; range, 369–1391 days) (Table 2). At final follow-up, posterior stability assessment demonstrated grade 0 laxity in 75% of patients and grade 1 laxity in the remaining 25%, as assessed by lateral stress radiographs (Figure 6 and Table 3). No graft failures, neurovascular complications, infections, or revision PCLR procedures were observed during the follow-up period. At final follow up, the mean knee range of motion was 140.0 ± 12.7 degrees (Table 3). Postoperative R.O.M. was 145.0° [interquartile range (IQR), 133.8–150.0°; range, 110–150°]. The median posterior tibial translation was 8.9 mm IQR, 8.0–9.4 mm; range, 2.1–11.5 mm, in the operated knee and 6.0 mm (IQR, 4.6–8.1 mm; range, 2.3–11.9 mm) in the contralateral knee. The median STSD was 2.7 mm (IQR, 1.7–3.2 mm; range, −2.7–4.8 mm). Two patients exhibited reduced knee flexion (<120 degrees). One patient (Patient No. 5), who had previously undergone revisional PCLR, developed postoperative arthrofibrosis and subsequently required arthroscopic arthrolysis followed by prolonged supervised physical therapy, ultimately achieving 120 degrees of knee flexion at final follow-up (Table 2). The second patient (Patient No. 10) demonstrated a maximum flexion of 110 degrees, which was attributed to excessive thigh circumference and was comparable to the contralateral limb (Table 3).

## 4. Discussion

This study describes and demonstrates the feasibility of a consistent, landmark-based arthroscopic technique for tibial tunnel placement in remnant-preserving PCLR. By employing the champagne-glass drop-off as a sagittal-plane guide and the lateral cartilage point as a coronal-plane reference, tibial tunnels were consistently positioned within the targeted inferolateral quadrant of the tibial PCL footprint while preserving maximal native ligament tissue. Postoperative MRI showed appropriate tunnel placement in all patients, with no tunnel-related complications, graft failures, neurovascular complications, infections, or revision PCLR procedures observed. At the final follow-up, patients demonstrated satisfactory functional outcomes, with a mean knee range of motion of 140.0° and posterior laxity limited to grade 1 or less. These findings underscore both the anatomic consistency and clinical feasibility of the proposed dual-landmark approach.

Regarding the biomechanical rationale for tunnel positioning, previous studies have emphasized the importance of inferior and lateral tibial tunnel placement. Cadaveric studies have demonstrated that anatomically inferior tibial tunnel positioning reduces posterior tibial translation compared with more proximal placements [10]. Similarly, lateral tunnel alignment has been associated with improved clinical outcomes, as it decreases graft bending stress and mitigates the “killer turn” phenomenon [6,7]. Our findings are consistent with this body of evidence (Supplementary Table S1 and Supplementary Figure S1). In a previous clinical series from our institution using only the champagne-glass drop-off as a reference, lateral tunnel positioning was achieved in approximately 78% of cases, whereas medial tunnel placement occurred in 22% of patients. Notably, posterior stability was significantly superior in the lateral placement group, with a nearly 3 mm reduction in the STSD of posterior tibial translation compared with the medial placement group (*p* = 0.0257).

Compared with previously described tunnel-positioning techniques, accurate localization of the tibial footprint during PCLR remains technically challenging, particularly in remnant-preserving procedures, because the tibial PCL footprint is deeply situated within the posterior compartment, arthroscopic visualization is frequently obscured by residual ligament tissue, and the proximity of the popliteal neurovascular structure limits aggressive debridement and instrument manipulation. Although fluoroscopic guidance and arthroscopic trans-septal techniques have improved tibial tunnel localization during PCLR, accurate identification of the ideal tibial footprint remains challenging because existing methods may still be affected by tibial rotation, overlapping osseous structures, soft tissue coverage, or remnant preservation [9,18]. In particular, previously described palpation-based techniques remain operator-dependent and may be less reliable when the foveal margins are obscured [9]. Ahn and Lee subsequently proposed distance-based methods for tibial tunnel localization using fixed centimeter- or millimeter-based references [9,10]. Nevertheless, these approaches do not account for interindividual variations in tibial morphology or PCL remnant thickness. Moreover, these techniques primarily provide guidance in the sagittal plane and do not adequately address coronal-plane (medial–lateral) tunnel positioning.

Bony landmarks may provide a more anatomically consistent reference framework for tibial tunnel localization during remnant-preserving PCLR. Bony landmarks provide fixed and reproducible reference points that are independent of soft tissue variability and intraoperative imaging quality, thereby offering greater reliability for anatomic guidance. The champagne-glass drop-off has been recognized as a dependable sagittal-plane bony landmark and can be consistently identified arthroscopically [8]. However, until recently, a reliable coronal-plane (medial–lateral) bony landmark for tibial tunnel placement in PCLR had not been validated. This lack of coronal-plane guidance likely contributes to the frequent medial deviation of tibial tunnels observed in clinical practice. In a previous clinical series from our institution that relied solely on the champagne-glass drop-off as a reference, tunnel placement within the inferolateral quadrant was achieved in 78% of cases, whereas inferomedial tunnel placement occurred in the other 22% of patients. The medial tunnel position was associated with less favorable posterior stability outcomes in that cohort. This finding may reflect the limitation of relying on a single sagittal-plane landmark, which primarily provides guidance in the sagittal-plane but does not allow precise control of medial–lateral variation. Anderson et al. used cadaveric dissections to precisely define the tibial attachment of the PCL bundles and identified the “lateral cartilage point,” located at the lateral margin of the bundle ridge, as the lateral boundary of the tibial footprint [12]. In the present study, integrating sagittal and coronal bony landmarks, tibial tunnel placement within the targeted inferolateral zone was demonstrated on postoperative MRI for all patients, demonstrating consistent tunnel positioning within this case series.

Remnant preservation represents another important consideration in modern PCLR techniques. Although Anderson et al. demonstrated that the lateral cartilage point can be visualized arthroscopically, their technique required extensive removal of native PCL tissue when accessed through anterior portals. In contrast, recent studies have increasingly emphasized the advantages of remnant-preserving PCLR, reporting favorable clinical and radiological outcomes [13]. Multiple investigations have confirmed the presence of mechanoreceptors within the PCL, which contribute to proprioception and play a critical role in maintaining knee joint stability and preventing secondary injury [14,15,16]. In addition, preserved PCL remnants may function as a biological cushion between the graft and bone, increasing the effective intra-articular graft length and improving graft isometry [17]. In the current study, we describe an arthroscopic technique that enables consistent identification of both the champagne-glass drop-off and the lateral cartilage point while preserving maximal native PCL tissue.

From a clinical perspective, this dual-landmark approach may offer several practical advantages beyond anatomical consistency. First, the proposed bony landmarks provide a relatively objective and anatomically consistent reference framework for tibial tunnel placement. Compared with soft tissue–based landmarks, these fixed osseous references may be less affected by remnant tissue or synovial variability and therefore may facilitate more consistent intraoperative localization across different surgeons and clinical settings. Second, because the procedure was performed without intraoperative fluoroscopy in this series, the technique may offer potential procedural advantages such as reducing operative time, radiation exposure, and contamination risk. Third, anatomically optimized inferolateral tunnel positioning combined with remnant preservation may enhance long-term graft durability by minimizing stress concentration and reducing the risk of graft elongation or failure. Although the direct relationship between tibial tunnel position and clinical outcomes remains an area of ongoing investigation, the consistency achieved with this dual-landmark method represents a meaningful step toward procedural standardization in PCLR. If widely adopted, this approach may facilitate more consistent comparisons of surgical outcomes across studies and institutions.

Several strengths support the validity of the present findings. All procedures were performed by a single experienced surgeon using a uniform surgical technique, minimizing technical variability. Postoperative rehabilitation was standardized and supervised by a single physical therapist, ensuring consistency in postoperative care. Importantly, objective postoperative magnetic resonance imaging was used to confirm tibial tunnel positioning, rather than relying solely on intraoperative assessment or radiographic imaging. Finally, clinical outcomes were assessed with an average follow-up of 2 years, allowing for short-term evaluation of graft function and knee stability.

Nevertheless, several limitations should be acknowledged. First, the sample size was relatively small (*N* = 20), reflecting the lower incidence of PCL injuries and challenges in complete follow-up during the COVID-19 pandemic. A total of 11 of 31 eligible patients were excluded because of incomplete postoperative MRI or clinical follow-up, resulting in a 35.4% loss to follow-up rate. A reliable comparison between included and excluded patients could not be performed because baseline and follow-up data were incomplete for the excluded patients. Therefore, the exclusion of 11 of 31 eligible patients may have introduced selection bias, and the final cohort may not fully represent the entire eligible population undergoing remnant-preserving PCLR with the dual-landmark technique. Although partly related to COVID-19–associated disruptions, future prospective studies with standardized follow-up and lower attrition rates are warranted. Second, tunnel position was assessed using MRI at 1 year postoperatively, using a predefined categorical classification system rather than quantitative coordinate-based measurements. Therefore, this method may not fully capture subtle variations in tunnel aperture position anatomic precision. Computed tomography performed immediately after surgery may provide more precise evaluation of tunnel orientation and aperture geometry, although radiation exposure and insurance limitations precluded routine postoperative CT in this cohort. Third, while tunnel placement was evaluated objectively with MRI, this study did not include biomechanical testing or long-term functional outcomes, and therefore the direct relationship between tunnel accuracy and long-term graft survival remains to be established. Fourth, this was a retrospective case series (Level IV evidence), and comparative studies are needed to confirm the superiority of this technique over existing methods. Fifth, MRI assessments were performed by investigators involved in the study rather than by independent external reviewers, which may introduce observer-related bias. In addition, the absence of interobserver variability in this study precluded formal statistical assessment of agreement. Although postoperative range of motion and posterior stability on stress radiographs were assessed, validated patient-reported outcome measures, including the IKDC subjective score, Lysholm score, Tegner activity scale, KOOS, and return-to-sport rate, were not consistently available because of the retrospective design of this study. Therefore, the present study cannot fully evaluate patient-perceived functional recovery or overall clinical success after PCLR. Future prospective studies incorporating validated functional scores and return-to-sport outcomes are warranted to assess more comprehensively the clinical effectiveness of this technique. Sixth, our study cohort included isolated, revisional, and combined PCLR procedures. Concomitant procedures such as ACL reconstruction and high tibial osteotomy may have influenced the postoperative rehabilitation, knee stability, range of motion, and other clinical outcomes. Consequently, the postoperative outcomes should be interpreted cautiously and cannot be solely attributed to the proposed technique. Seven, although no tunnel related complications, graft failures, neurovascular complications, infections, or revision PCLR procedures were observed in this series, the relatively small sample size and retrospective design limit definitive conclusions regarding the safety and generalizability of the technique. Larger multicenter studies with longer follow-up are required to further validate these findings. Finally, although complete agreement was observed between the two MRI evaluators, this reflects only postoperative MRI-based tunnel classification and does not establish reproducibility of intraoperative landmark identification among surgeons. Future multi-surgeon studies are needed to assess the reproducibility of this technique.

Future investigations should focus on prospective, randomized controlled trials comparing this dual-landmark approach with fluoroscopic-guided or palpation-based techniques. Biomechanical modeling and finite element analyses may further elucidate the effects of inferolateral tunnel positioning on graft stress distribution under dynamic loading. Long-term follow-up studies extending beyond 5 to 10 years are essential to determine whether improved tunnel accuracy translates into superior graft longevity and reduced osteoarthritic progression. Additionally, the applicability of this technique should be evaluated in complex scenarios, including multi-ligamentous knee injuries and revision PCLR, where anatomical distortion is more pronounced.

## 5. Conclusions

This study describes a dual-landmark arthroscopic technique for tibial tunnel placement in remnant-preserving PCLR using readily identifiable arthroscopic bony landmarks, namely the champagne-glass drop-off and the lateral cartilage point. The procedure was performed without the use of intraoperative fluoroscopy. In this case series, postoperative MRI demonstrated consistent tunnel positioning within the targeted inferolateral region of the PCL tibial footprint. No tunnel-related complications, graft failures, neurovascular complications, infections, or revision PCLR procedures were observed for all patients during follow-up. One patient with a history of prior revisional PCLR underwent arthroscopic arthrolysis for postoperative arthrofibrosis, which was considered a stiffness-related postoperative complication rather than a tunnel-related complication. Postoperative stability and range of motion were satisfactory in this case series. These findings support the clinical feasibility and anatomic consistency of the proposed technique. While success in PCLR depends on multiple factors, this method may help facilitate one of the most technically challenging steps and may facilitate more standardized intraoperative localization of tibial tunnel in future surgical practice. Future multicenter and long-term studies will be necessary to evaluate its generalizability and broader clinical applicability. In addition, multi-surgeon studies will be performed to assess the reproducibility of this technique.

## Figures and Tables

**Table 1:** General Characteristics and MRI Evaluation.

Number	Sex	Age at Operation	Type of Graft ^a^	Graft Size (mm)	Surgery ^b^	MRI Evaluation (by Author 1)		MRI Evaluation (by Author 2)	
						Sagittal	Coronal	Sagittal	Coronal
1	F	40	Auto peroneal longus + semi-T	9	PCLR	inferior	lateral	inferior	lateral
2	M	29	Allo Achilles	9.5	PCLR	inferior	lateral	inferior	lateral
3	M	16	Auto peroneal longus + semi-T	9	PCLR	inferior	lateral	inferior	lateral
4	M	26	Auto Semi-T + gracilis	8	PCLR	inferior	lateral	inferior	lateral
5	F	23	Auto Semi-T + gracilis	9	Revision PCLR	inferior	lateral	inferior	lateral
6	M	39	Auto Peroneal longus + semi-T	9.5	PCLR + HTO	inferior	lateral	inferior	lateral
7	M	38	Auto Semi-T + gracilis	9	PCLR	inferior	lateral	inferior	lateral
8	M	41	Auto Peroneal longus	8	PCLR	inferior	lateral	inferior	lateral
9	M	17	Allo Achilles	9	PCLR	inferior	lateral	inferior	lateral
10	M	25	Auto Peroneal longus + semi-T	9.5	PCLR	inferior	lateral	inferior	lateral
11	F	19	Allo Achilles	8.5	PCLR + ACLR	inferior	lateral	inferior	lateral
12	M	22	Auto Peroneal longus + 4HT	9	PCLR	inferior	lateral	inferior	lateral
13	M	19	Allo Achilles	10	PCLR	inferior	lateral	inferior	lateral
14	F	23	Auto Peroneal longus + Gracilis	9	PCLR + PLCR	inferior	lateral	inferior	lateral
15	F	28	Auto Peroneal longus + semi-T	8.5	PCLR	inferior	lateral	inferior	lateral
16	M	22	Allo Achilles	9.5	PCLR + PLCR	inferior	lateral	inferior	lateral
17	M	36	Allo Achilles	10	PCLR	inferior	lateral	inferior	lateral
18	M	30	Allo Achilles	9.5	PCLR	inferior	lateral	inferior	lateral
19	M	21	Auto Peroneal longus + semi-T	9	PCLR	inferior	lateral	inferior	lateral
20	F	26	Allo Achilles	9	PCLR + HTO	inferior	lateral	inferior	lateral

^a^ Auto, autogenous; Allo, allogenous. ^b^ PCLR, posterior cruciate ligament reconstruction; ACLR, anterior cruciate ligament reconstruction; PLCR, posterolateral complex reconstruction; HTO, high tibial osteotomy.

**Table 2:** Complications and Follow-up Duration.

Number	Complication/Reoperation	Follow-Up Duration (Days)
1	no	384
2	no	880
3	no	1314
4	no	714
5	Arthrofibrosis/Arthroscopic arthrolysis	761
6	no	877
7	no	1391
8	no	760
9	no	841
10	no	935
11	no	672
12	no	379
13	no	723
14	no	775
15	no	729
16	no	369
17	no	814
18	no	730
19	no	392
20	no	544

**Table 3:** Postoperative Clinical Outcomes at final follow-up: Range of Motion, Laxity.

Number	R.O.M (°)	Pre-OP-Posterior Instability (Grade) ^a^	Post-OP-Posterior Instability (Grade) ^a^	Operation Knee (mm) ^b^	Opposite Knee (mm) ^b^	STSD (mm) ^c^
1	145	2	0	7.6	5.1	2.5
2	150	2	0	9.2	11.9	−2.7
3	150	2	1	8.1	3.5	4.6
4	150	3	0	9.4	6.6	2.8
5	120	2	0	9.5	6.7	2.8
6	125	3	0	11.5	8.9	2.6
7	150	2	0	9.0	8.1	0.9
8	150	3	1	9.2	4.8	4.4
9	150	3	1	8.9	4.6	4.3
10	110	2	0	5.7	3.9	1.8
11	150	2	1	8.7	3.9	4.8
12	145	2	0	9.7	8.2	1.5
13	150	2	0	10.7	9.2	1.5
14	120	2	0	7.5	5.8	1.7
15	150	2	0	10.9	8.1	2.8
16	145	3	0	8.2	6.1	2.1
17	135	2	0	2.1	2.3	−0.2
18	135	2	0	8.1	6.3	2.8
19	140	2	0	7.6	4.9	2.7
20	130	2	1	8.8	4.5	4.3

^a^ Pre-OP, pre-operation; Post-OP, post-operation. ^b^ The distance of posterior translation measured on stress films. ^c^ STSD, side-to-side difference.

## Data Availability

The datasets generated and/or analyzed during the current study are available from the corresponding author upon reasonable request, subject to institutional regulations and data protection policies.

## Supplementary Materials

The following supporting information can be downloaded at: https://www.mdpi.com/article/10.3390/diagnostics16111688/s1, Figure S1: This bar chart shows the comparison of mean STSD between patients with medially placed tibial tunnel and laterally placed ones from the previous data; Table S1: The result of MRI evaluation of the previous data; Video S1: This video embedded in PPT slide represents how to recognize the lateral border.

## Abbreviations

The following abbreviations are used in this manuscript:

PCL	Posterior cruciate ligament
PCLR	Posterior cruciate ligament reconstruction
ACL	Anterior cruciate ligament
PLCR	Posterolateral complex reconstruction
