# Supplementary material for: A Consistent Landmark for Tibial Tunnel Placement in Arthroscopic Remnant-Preserving Posterior Cruciate Ligament Reconstruction: Use of Champagne-Glass Drop-Off and Lateral Cartilage Point—A Retrospective Case Series
Source: Diagnostics (Basel). 2026 May 29;16(11):1688. doi: 10.3390/diagnostics16111688 (PMC13257378; doi:10.3390/diagnostics16111688)
Supplement: Supplementary file 1 [file diagnostics-16-01688-s001.zip › diagnostics-4300042-supplementary.pdf]

Supplementary materials

Materials and Methods

This retrospective study reviewed consecutive patients who underwent single-bundle PCLR performed by a single orthopedic surgeon between Jan 2018 and Feb 2020 at Chung Shan Medical University Hospital, Taichung, Taiwan. The inclusion and exclusion criteria were as follows.

Inclusion criteria:

- Patients who underwent remnant-preserving single-bundle PCLR
- Availability of complete postoperative clinical follow-up
- Availability of postoperative MRI

Exclusion criteria:

- Incomplete clinical follow-up
- Incomplete MRI follow-up

All surgical procedures were performed by the same surgeon using a standardized arthroscopic technique (trans-septal portal remnant preserving PCLR with using only Champagne-Glass Drop-Off landmark) and uniform postoperative rehabilitation protocol.

Results

Supplementary Table S1. The result of MRI evaluation of the previous data. The data was collected from 27 patients underwent PCLR with only using champagne-glass drop-off as landmark.

		Number of cases
Sagittal	Inferior	27
	Superior	0
Coronal	Lateral	21
	Medial	6

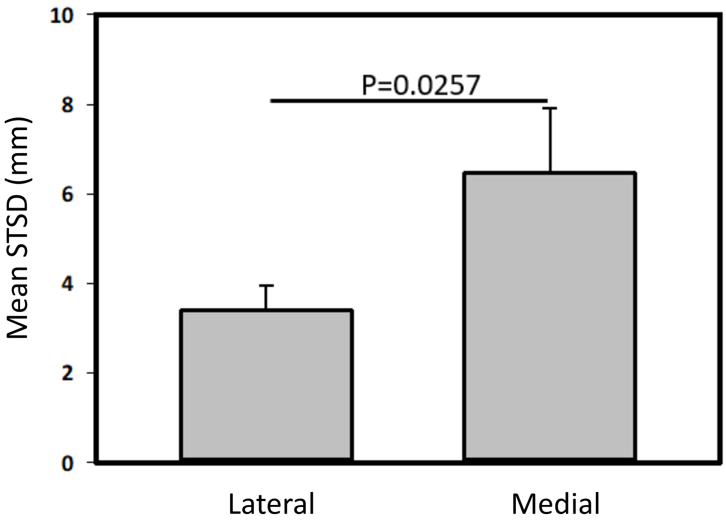

Supplementary Figure S1. This bar chart shows the comparison of mean STSD between patients with medially placed tibial tunnel and laterally placed ones from the previous data. The data is collected from 27 patients underwent PCLR with only using champagne-glass drop-off as landmark.
