# Supplementary material for: A Consistent Landmark for Tibial Tunnel Placement in Arthroscopic Remnant-Preserving Posterior Cruciate Ligament Reconstruction: Use of Champagne-Glass Drop-Off and Lateral Cartilage Point—A Retrospective Case Series
Source: Diagnostics (Basel). 2026 May 29;16(11):1688. doi: 10.3390/diagnostics16111688 (PMC13257378; doi:10.3390/diagnostics16111688)
Supplement: Supplementary file 1 [file diagnostics-16-01688-s001.zip › video .pptx]

## Slide 1
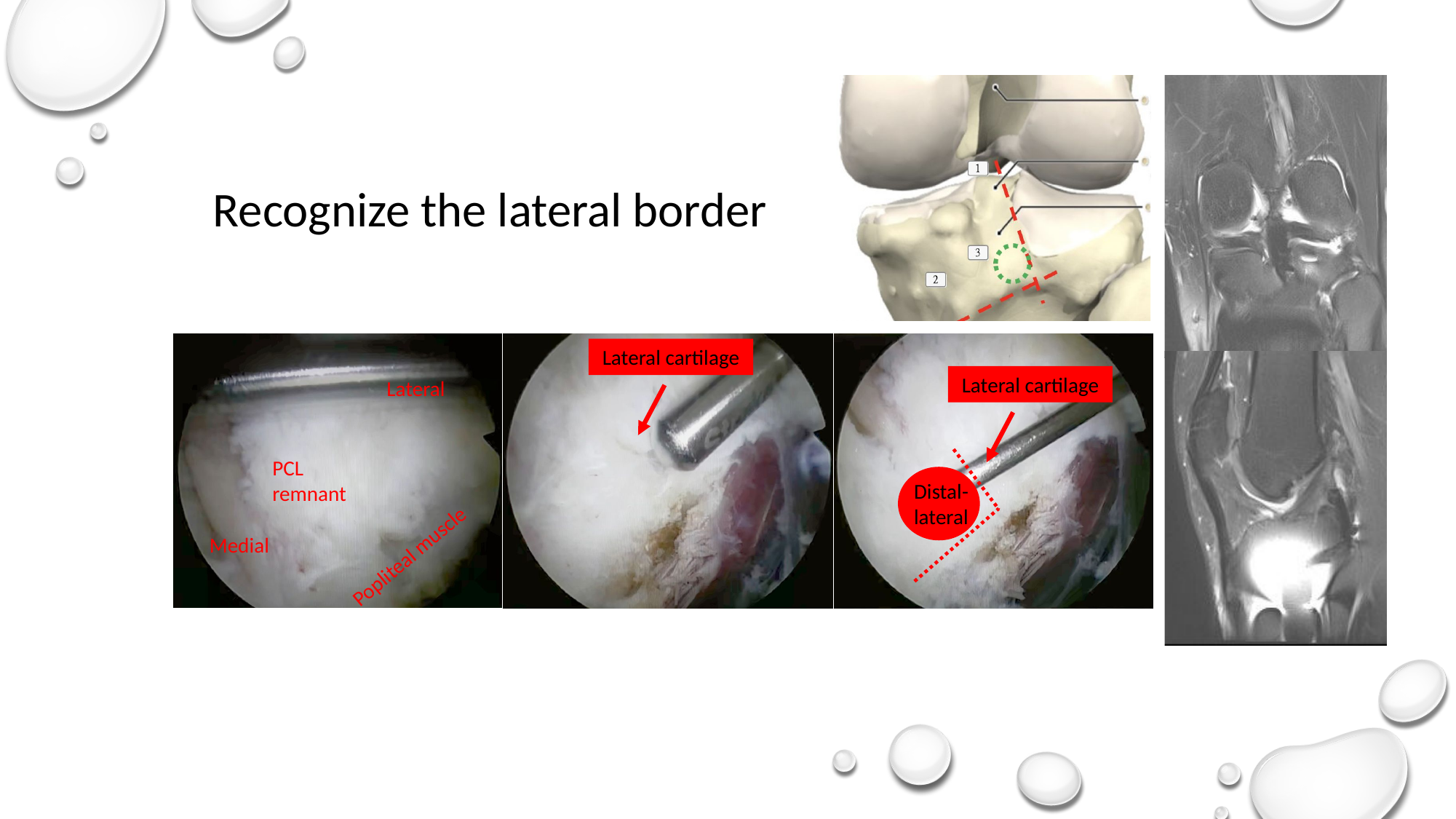

Recognize the lateral border
Lateral cartilage
Lateral cartilage
Lateral
PCL remnant
Distal- lateral
Medial
Popliteal muscle
